# The immune evasion ability of Delta variant is comparable to that of Beta variant in South Africa

**DOI:** 10.1186/s12889-023-15431-2

**Published:** 2023-03-17

**Authors:** Daihai He, Boqiang Chen, Shi Zhao, Lewi Stone

**Affiliations:** 1 Department of Applied Mathematics, Hong Kong Polytechnic University, Hong Kong, China; 2 Research Institute for Future Food, The Hong Kong Polytechnic University, Hong Kong, China; 3 JC School of Public Health and Primary Care, Chinese University of Hong Kong, Hong Kong, China; 4 CUHK Shenzhen Research Institute, Shenzhen, China; 5 Mathematical Sciences, School of Science, RMIT University, Melbourne, Australia; 6 Biomathematics Unit, School of Zoology, Faculty of Life Sciences, Tel Aviv University, Tel Aviv, Israel

**Keywords:** Immune evasion, Beta variant, Delta variant, Reinfection, South Africa

## Abstract

**Background:**

The high immune evasion ability of SARS-COV-2 Omicron variant surprised the world and appears to be far stronger than any previous variant. Previous to Omicron it has been difficult to assess and compare immune evasion ability of different variants, including the Beta and Delta variants, because of the relatively small numbers of reinfections and because of the problems in correctly identifying reinfections in the population. This has led to different claims appearing in the literature. Thus we find claims of both high and low immune evasion for the Beta variant. Some findings have suggested that the Beta variant has a higher immune evasion ability than the Delta variant in South Africa, and others that it has a lower ability.

**Method:**

In this brief report, we re-analyse a unique dataset of variant-specific reinfection data and a simple model to correct for the infection attack rates of different variants.

**Result:**

We find that a model with the Delta variant having  an equal or higher immune evasion ability than Beta variant is compatible with the data.

**Conclusion:**

We conclude that the immune evasion ability of Beta variant is not stronger than Delta variant, and indeed, the immune evasion abilities of both variants are weak in South Africa.

**Supplementary Information:**

The online version contains supplementary material available at 10.1186/s12889-023-15431-2.

## Background

Rates of COVID-19 reinfections, through both immunity evasion and waning immunity, have proven difficult to assess with consistency. Nevertheless, it is crucial to understand the different immune evasion abilities of previous SARS-CoV-2 variants to help us interpret the high immune evasion ability of Omicron and future variants. Shinde et al. and Madhi et al. [1, 2] argued that previous COVID-19 infection provides little protection against reinfection by the Beta (B.1.351) variant in South Africa. Shinde et al. suggested this needs further future study and considered the outcome as preliminary results. Lack of protection was also suggested with model-based estimates of immune evasion at 63.4% for the Beta variant and 24.5% for the Delta (B.1.617.2) variant [3]. In contrast, Chemaitelly [4] demonstrated that previous infection induced strong protection against infection with  the Beta variant, as high as 92.3% in Qatar. Similarly, Pulliam et al. [5] could find no evidence of immunity evasion associated with Beta and Delta variants. Because of these differences, the recent detailed household study in South Africa of Cohen et al. [6] provides helpful new details on reinfection rates of Beta and Delta variants, which we seek to re-examine with a more refined analysis.

Over the study period July 2020 to August 2021, South Africa experienced three COVID-19 waves caused by the ancestral strain, the Beta variant and the following Delta variant [5, 6]. Cohen et al. found that 749 of 1200 individuals (62.4%) had at least one SARS-CoV-2 infection, and 87 of 749 (11.6%) were reinfected (including probable, possible and confirmed reinfections) [6]. However, Cohen et al. [6] did not compare the different reinfection abilities of the Beta and Delta variants. Based on Fig. 3 of Ref [6], we provide a summary of different reinfection types in Table S1 (first column). The most frequent reinfection is of the Beta-Delta type (43 occurrences), i.e., a person infected with the Beta variant later becomes infected with Delta variant. The second and third most frequent reinfection types are Ancestral-Delta type (20 occurrences) and the Ancestral-Beta type (17 occurrences). The data thus already tends to indicate that Delta leads to more reinfections than Beta, although it is difficult to be sure without a proper analysis. We made an assumption that if a seroconversion occurs in the period a variant dominates, then it is associated with an infection with the variant. This type of variant-specific data is unique to the best of our knowledge.

## Method

Following [5], we correct for infection attack rate and susceptible pool size to assess immune evasion of Beta and Delta variants. This is based on the fact that the Delta variant, which arrived later, has a larger pool of previously infected it can reinfect, an effect that needs to be factored out. We denote reported cumulative infections of Ancestral (A), Beta (B), and Delta (D) variants as ${c}_{1}$, ${c}_{2}$, and ${c}_{3}$, respectively, which can be approximated by the reported cumulative infections in three time-intervals, which are bounded by the timings that the Beta variant became dominant and the Delta variant became dominant and by the end of the study, i.e., August 28, 2021 [6]. The Beta variant exceeded 50% of all samples sequenced in South Africa in the week of Oct 26, 2020 and the Delta variant in the week of June 14, 2021 [7].

We let AB represent the number of individuals infected by A and then reinfected by the B strain etc. The naïve relative frequencies of observing AB, BD and AD reinfections are ${c}_{1}{c}_{2}$: ${c}_{2}{c}_{3}$: ${c}_{1}{c}_{3}.$ But note  that the relatively longer delay between A infection wave and D infection wave would mean more natural waning of protection for those previously infected with A, and thus a relatively higher frequency of AD reinfections than AB reinfection. Thus, we include a factor to the ${c}_{1}{c}_{3}$ term, i.e., ${c}_{1}{c}_{2}$: ${c}_{2}{c}_{3}$: ${bc}_{1}{c}_{3}$, where $b\in [1, 2]$ is a reasonable first approximation [8] that we can explore. There are two cases to consider:

- Case 1: If the immune evasion of Beta is $\alpha$-fold ($\alpha \in [1, 2]$) of the Delta variant, then the relative frequency becomes ${\alpha c}_{1}{c}_{2}$:${c}_{2}{c}_{3}$:${bc}_{1}{c}_{3}$.
- Case 2: If the immune evasion of Delta is $\alpha$-fold ($\alpha \in [1, 2]$) of the Beta variant, then the relative frequency becomes ${c}_{1}{c}_{2}$:$\alpha {c}_{2}{c}_{3}$:${bc}_{1}{c}_{3}$.

## Results

**Fig. 1:**
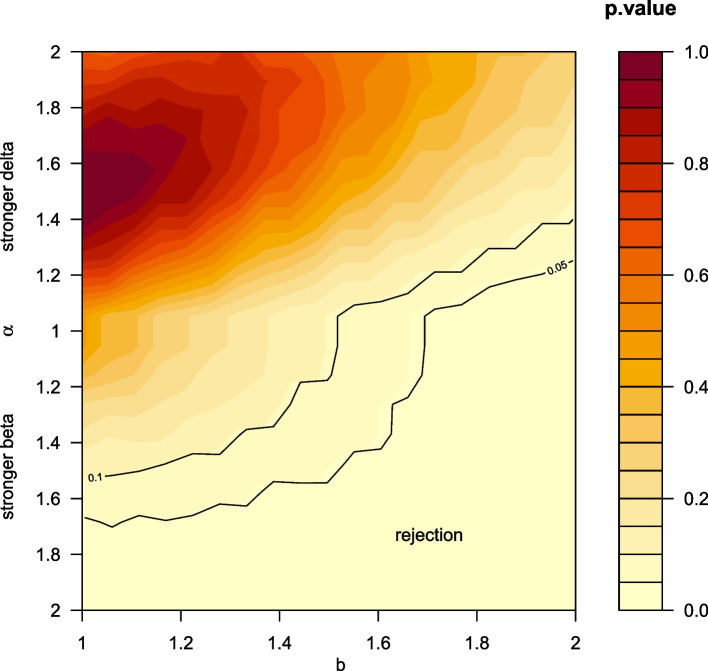
Given the observed frequencies of different types of reinfections from Ref [6], we calculate the p-value under different hypothesized models with different value of $\alpha ,b$ using the ${\chi }^{2}$(chi-squared) test (see SI1). The rejection region falls in the region of “stronger Beta immune evasion” scenario

We add the 4 BB reinfections into the 17 AB infections) to obtain 21 reinfections that we term AB, caused by the Beta variant. For AB, BD and AD, the observed frequency is 21, 43, 20, and the total number of reinfections is 84. Other types of reinfections have been ignored due to low number of observed frequencies. Given the total number of reinfections is 84, the theoretical frequencies, the test statistic and *p*-value (${\chi }^{2}$-test, see supplementary SI1) can be calculated for different values of $\alpha$. In Fig. 1, we show the p-value as function of values of alpha (y-axis) and b (x-axis).

We show two curves of *p*-value = 0.1 and 0.05. It is very clear that the large rejection region falls in the “stronger Beta” region. A stronger Beta immune escaping (relative to Delta) conclusion is thus not supported by these data (the p-value is less than *p* = 0.05)  On the contrary, a stronger Delta immune escaping (relative to Beta) conclusion is favored by these data. We give more explanation in the supplementary text and Table S1.

After correction for the different infection attack rates of the variants, the Delta variant still has a comparable, if not stronger, immune evasion ability than the Beta. This result conflicts with modelling estimates in Ref. [3], which conclude Beta variants have the stronger immune evasion ability.

## Discussion

Our work is interesting in the way we performed a head-to-head comparison between Beta and Delta. Not only were we unable to find evidence to support a stronger immune escaping ability for Beta than Delta, but also we found Delta likely had a stronger immune escaping ability than Beta. Furthermore, if we consider the overall reinfection ratio is 11.6% after three waves of infections [6], the immune escaping ability of both Beta and Delta are weak in this household study. Pulliam et al. [5] found no immune escaping for Beta and Delta in South Africa in a large-scale data analysis. These findings are in line with [4, 9]. All these studies suggested weak immune escaping for Beta and Delta.

The studies that suggested strong immune escaping for Beta and Delta should be viewed as preliminary observations since they were not designed for the purpose of estimating immune evasion [1, 2] and/or have wide confidence intervals [3]). Dhar et al. [10] estimated “a 27.5% reinfection rate during the Delta pandemic wave in Delhi, India” which was cited by [3]. However, it was recently found that the methodology of [10] could suffer overfitting, thus the estimate is questionable [11].

We elaborate our methodology in supplementary SI1 and the data collection and limitation in SI2. In summary, we conclude that our head-to-head comparison study found Delta had a comparable if not stronger immune escaping ability than Beta in South Africa.

## Supplementary Information

**Additional file 1: Table S1. **Summary of different type ofreinfections from Ref [3].

## Data Availability

Data are in the main text and from Figure 3 of Ref 6.
