# Supplementary material for: The immune evasion ability of Delta variant is comparable to that of Beta variant in South Africa
Source: BMC Public Health. 2023 Mar 17;23:511. doi: 10.1186/s12889-023-15431-2 (PMC10020070; doi:10.1186/s12889-023-15431-2)
Supplement: Supplementary file 1 — Additional file 1: Table S1. Summary of different type ofreinfections from Ref [3]. [file 12889_2023_15431_MOESM1_ESM.docx]

The immune evasion ability of Delta variant is comparable to that of Beta variant in South Africa

Daihai He^1,2^, Boqiang Chen^1^, Shi Zhao^3,4^, Lewi Stone^5,6^

**Supplementary**

SI1. Calculation of relative frequency:

It is reasonable to assume a person’s risk of infection with a variant in a period is proportional to the total reported cases in the same period. If a disease is rarely (/frequently) reported, then a random person’s probability of infection will be low (/high). We further assume a person’s risk of double infection with two variants is proportional to the product of the total reported cases or deaths due to the two variants and the immune escaping ability of the later variant against infection of the former variant. We use the total reported cases in the time interval when a variant dominated to approximate the total infection of the variants. We could consider the reporting ratio (due to under reporting), but as long as the reporting ratio is relatively constant, it will have no impact on the calculation of a relative frequency.

We assume two diseases dominate in turn in a time period among a population.

We call these two diseases, disease A and disease B. We assume that disease A infected c1% of the population and disease B infected c2% of the population. Under ideal (homogenous) situation, a typical person from this population infected with both would be c1%* c2%. In our case, we have mainly three variants, i.e., Ancestral, Beta and Delta dominated in turn in our case. Thus, there are three types of reinfections: Ancestral-Beta, Ancestral-Delta, and Beta-Delta. We assume each of the variants infected, c1%, c2%, c3%. Thus the relative frequency of the three types of reinfections would be c1%*c2% : c2%*c3% : c1%*c3%. However, the mean time gap between Ancestral wave and Delta wave is double that of the Ancestral wave and the Beta, and a longer time gap means higher risk of reinfection for Ancestral-Delta than Ancestral-Beta.

Thus, a factor b∈[1,2] is inserted to account for this, such that the relative frequency is c1%*c2% : c2%*c3% : b*c1%*c3%. The factor represents the relative fold change between Ancestral-Delta than Ancestral-Beta, due to a long gap between Ancestral-Delta than Ancestral-Beta.

We used the total reported cases in South Africa during the three time -intervals when Ancestral, Beta and Delta dominate (their proportion of sequenced sample >50% for a variant) in South Africa to approximate c1%, c2%, and c3%. In the above, we assume equal immune evasion ability for Beta and Delta variant. We consider a factor α∈[1,2] to account for the relative fold change due to higher immune escaping ability of Beta than Delta, or vice versa (if Beta stronger than Delta, then α is multiplied on Beta term. otherwise, α is multiplied on Delta term).

When Beta has a relatively stronger immune escaping ability than Delta, the relative frequency of reinfections would be

α* c1%*c2%, c2%*c3%, and b*c1%*c3%.

When Delta has a relatively stronger immune escaping ability than Beta, the relative frequency would be

c1%*c2%, α*c2%*c3%, and b*c1%*c3%.

The three time intervals for Ancestral, Beta and Delta dominance are between 2020-3-1 and 2020-10-26, between 2020-10-26 and 2021-06-14, and between 2021-06-14 and 2021-08-28, respectively, according to variant sequencing data in GISAID[1, 2].

We perform $\chi^{2}$ goodness-of-fit test. $O_{i}$ and $E_{i}$ denote the observed frequency and hypothesize frequency, respectively. Then $\chi^{2}=\sum_{i=1}^{3} \frac{{(O_{i}-E_{i})}^{2}}{E_{i}}$*.* We calculate the p-value which is the probability under the null hypothesis (ie, the theoretical frequency is true) to observe the observed frequency (or as extreme as the observed frequency) by chance.

Additional example:

Table S1. Summary of different type of reinfections from Ref [3]. We show the observed frequency in the first column, and four different hypothesized scenarios: 1&2 under stronger immune evasion for Beta variant, 3: equal immune evasion for Beta and Delta, and 4: stronger immune evasion for Delta variant. $\alpha=2,$ $\alpha=1.7,$ $\alpha=1,$ in the case 1 ${\alpha c}_{1}c_{2}$ :$c_{2}c_{3}$: ${1.5c}_{1}c_{3},$and $\alpha=1.5$ in the case 2 $c_{1}c_{2}$ :$\alpha c_{2}c_{3}$: ${1.5c}_{1}c_{3}$, as hypothesized scenarios 1 to 4. Chi-squared test p-value in last row shows that the first two scenarios is rejected, while the last two scenarios cannot be rejected.

| Type of reinfection | Observed frequency | Hypothesized 1 | Hypothesized 2 | Hypothesized 3 | Hypothesized 4 |
| --- | --- | --- | --- | --- | --- |
| Beta-Delta | 43 | 24 | 26 | 31 | 39 |
| Ancestral-Delta | 20 | 25 | 27 | 32 | 27 |
| Ancestral-Beta | 17 | 35 | 31 | 22 | 18 |
| Beta-Beta | 4 |  |  |  |  |
| Ancestral-Alpha | 1 |  |  |  |  |
| Ancestral- Ancestral | 1 |  |  |  |  |
| Delta-Delta | 1 |  |  |  |  |
| $\chi^{2}$ |  | 9.4436 | 7.154 | 4.7327 | 1.4684 |
| p-value |  | 0.0088 | 0.028 | 0.094 | 0.48 |

SI2. Data collection and limitation of [3]:

“Midturbinate nasal swabs, COVID-19 related symptoms, and health-seeking data were collected or recorded twice a week, and serum was collected every 2 months to measure SARS-CoV-2 antibodies, with a total of seven serum collections at each site.” “The study included 58 weeks of follow-up at the rural site (July 16, 2020 to Aug 28, 2021) and 56 weeks of follow-up at the urban site (July 27, 2020, to Aug 28, 2021).” “Of 1251 eligible household members in 222 households, 1200 (95·9%; 643 in the rural community and 557 in the urban community) were included in the analysis”.

The data collection [3] has limitations, e.g. selection bias and possible underreporting of infections. But the study has good sample size in this kind of study covering three wave, and frequent RT-PCR test and serological test are frequent. All these measures makes the impact of the selection bias and the chance of underreporting to be low.

1. Shu Y, McCauley J: **GISAID: Global initiative on sharing all influenza data–from vision to reality**. *Eurosurveillance* 2017, **22**(13):30494.

2. **CoVariants: SARS-CoV-2 Mutations and Variants of Interest.** [<https://covariants.org>]

3. Cohen C, Kleynhans J, von Gottberg A, McMorrow ML, Wolter N, Bhiman JN, Moyes J, du Plessis M, Carrim M, Buys A: **SARS-CoV-2 incidence, transmission, and reinfection in a rural and an urban setting: results of the PHIRST-C cohort study, South Africa, 2020–21**. *The Lancet Infectious Diseases* 2022.
